# Changing molecular conjugation with a phenazine acceptor for improvement of small molecule-based organic electronic memory performance†

**DOI:** 10.1039/c7ra11932a

**Published:** 2018-01-03

**Authors:** Quan Liu, Caibin Zhao, Guanghui Tian, Hongguang Ge

**Affiliations:** Shaanxi Province Key Laboratory of Catalytic Foundation and Applications, School of Chemical and Environmental Science, Shaanxi University of Technology Hanzhong 723001 China liuq@snut.edu.cn +86 916 2641660

## Abstract

Two conjugated small molecules with different molecular conjugation, 4′,4′′-(diazene-1,2-diyl)bis(2′,3′,5′,6′-tetrafluoro-*N*,*N*-diphenyl-[1,1′-biphenyl]-4-amine) (TPA-azo-TPA) and 4,4′-(perfluorophenazine-2,7-diyl)bis(*N*,*N*-diphenylaniline) (TPA-ph-TPA), in which the electron donor triphenylamine moiety is bridged using different electron-accepting azobenzene or phenazine blocks, were designed and synthesized. The TPA-ph-TPA molecule with a larger conjugation acceptor regularly formed a nanocrystalline film and the as-fabricated memory devices exhibited outstanding non-volatile write once read many (WORM) memory effects with an ON/OFF ratio ten times higher than that of TPA-azo-TPA. Using theoretical calculations, it was speculated that the memory performance is a result of an electric field induced charge transfer effect and the enhanced device performance of the acceptor molecular conjugation is because of the presence of a strong charge transfer effect. The experimental findings suggest that the strategy of molecular conjugation may promote the performance of small molecule-based organic electronic memory devices by an enhanced a strong charge transfer effect.

## Abstract 2

Two small molecules with electron-accepting azobenzene or phenazine blocks, were synthesized. The experimental findings suggest that the molecule with larger conjugation may promote the memory performance by an enhanced strong charge transfer effect.

## Graphical abstract

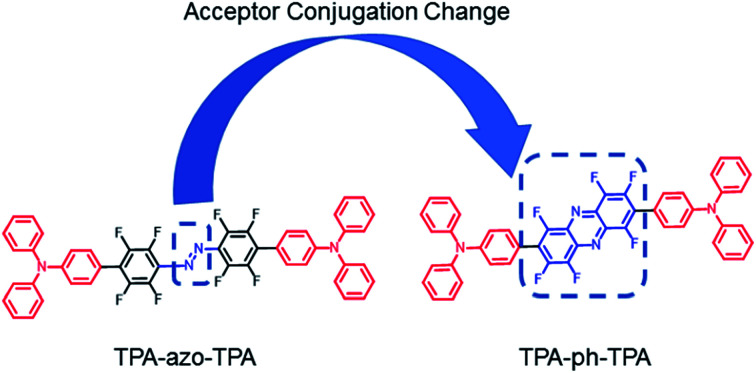

## Introduction

Traditional magnetic or optical material based data storage systems were limited by their memory capacity and cell size, which greatly restricted their use in applications for high density data storage.^1–3^ Organic electronic memory (OEM) is a promising alternative to achieve high-performance data storage and has gained significant scientific interest over the past few years. Many materials can be used for OEM, including polymeric materials,^4,5^ composite materials^6–8^ and small molecules.^9–11^ Among them, the organic small molecules have evoked considerable research interest because of their low cost, well-defined molecular structure, easy purification, and optoelectrical property tunability.^12^

Recently, numerous high-performance organic semiconductors and various outstanding memory behaviors have been successfully achieved using innovative molecular designs. Yam *et al.* designed a series of subphthalocyanin-based and boron(iii) diketonate-based molecules with tunable memory device performance.^13,14^ In previous work, the strength of push–pull electron groups was changed and this lead to the “charge trapping” hypothesis. Subsequently, tuning the length of the flexible alkyl side chain, molecular planarity, and conjugation length, was studied precisely to adjust the memory performances.^15–17^ Azobenzene (azo) because of its week electron withdrawing ability was commonly used for the “charge trapping” group in small molecule memory material.^18–20^ However, the stability and conjugate planarity of azobenzene are poor, which greatly limits their use because of their data storage performance. Phenazine (ph) derivatives play an important role in organic semiconductors for their wide range of photoelectric properties and stability.^21–23^ Therefore, it is important to design a new phenazine-based conjugate skeleton rather than one based on azobenzene.

**Scheme 1:**
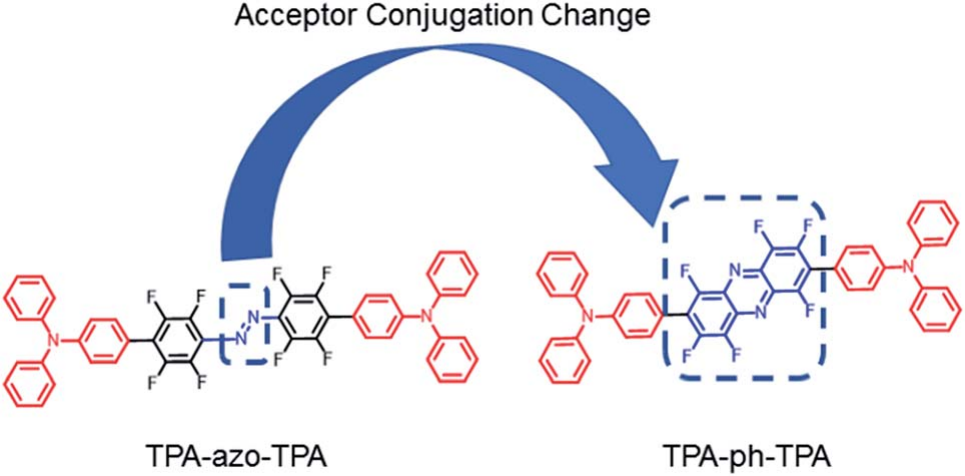
Molecular structures of the TPA-azo-TPA and TPA-ph-TPA.

**Scheme 2:**
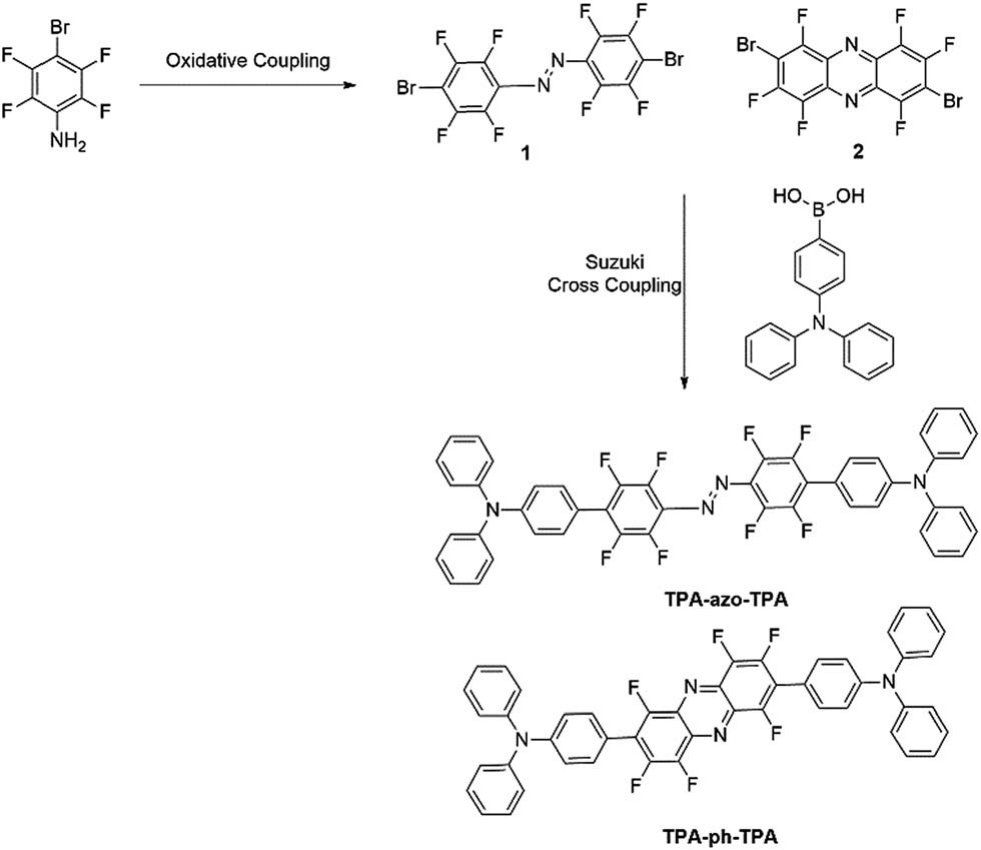
Synthesis routes of TPA-azo-TPA and TPA-ph-TPA.

In this work, first molecules linked with azobenzene (TPA-azo-TPA) were synthesized, and then the azobenzene was replaced with phenazine (TPA-ph-TPA), as shown in Scheme 1. Both molecules have an electron-donating triphenylamine moiety, which is bridged by blocks with different conjugation abilities. The influences of molecular conjugation changed the film morphology, and the memory device performance were precisely and systematically investigated. The results showed that the sandwich memory device with the larger conjugation molecule (TPA-ph-TPA) as the active layer exhibited better binary electrical conductance switching and nonvolatile memory effects with long-term thermal stability than the smaller conjugation molecule (TPA-azo-TPA) (Scheme 2).

## Experimental

### Materials

4-Bromo-2,3,5,6-tetrafluoroaniline, hypochlorous acid *tert*-butyl ester (*t*-BuOH), [4-(diphenylamino)phenyl]boronic acid, palladium diacetate, sodium iodide (NaI), potassium carbonate, [1,1′-bis(diphenylphosphino)ferrocene]dichloropalladium(ii) and potassium acetate were purchased from commercial sources (TCI, Alfa Aesar, and Sigma-Aldrich). All solvents were purchased from Sinopharm Chemical Reagent Co., Ltd. All chemicals were used as received without further purification.

### Characterization

All electrical measurements of the devices were performed under ambient conditions without any encapsulation using an HP 4145B semiconductor parameter analyzer (Hewlett Packard) equipped with an HP 8110A pulse pattern generator (Hewlett Packard). Nuclear magnetic resonance (NMR) spectra were recorded using an Inova 400 MHz Fourier transform (FT)-NMR spectrometer (Varian). High-resolution mass spectra were acquired using a Micromass GCT-time-of-flight (TOF) gas chromatography/mass (GC/MS) spectrometer (Waters) with an electrospray ionization source. The film thickness was determined using a Form Talysurf Intra Profiler (Taylor Hobson). Ultraviolet-visible (UV-vis) absorption spectra were measured at room temperature using a UV-3600 spectrophotometer (Shimadzu). Atomic force microscopy (AFM) measurements were performed using a MFP-3DTM AFM instrument (Digital Instruments/Asylum Research). Thermogravimetric analysis (TGA) was conducted using a Dynamic Rate method with a TGA 2950 (TA Instruments) at a heating rate of 10 °C min^−1^ under a nitrogen (N_2_) flow rate of 50 mL min^−1^.

### Fabrication of memory devices

The indium tin oxide (ITO) substrates were pre-cleaned sequentially with ethanol, acetone and isopropanol in an ultrasonic bath for 20 min. The benzene solution of TPA-ph-TPA and TPA-azo-TPA was filtered through poly(tetrafluoroethylene) membrane micro-filters with a pore size of 0.32 μm. Then, 0.1 mL of solution was spin coated onto the substrates at a spinning speed of 500 rpm for 10 s and then 2000 rpm for 30 s, followed by vacuum drying at 80 °C for 8 h. The thickness of the films was about 90 nm as measured using a model M2000 DI spectroscopic ellipsometer (J. A. Woollam, USA). To construct the aluminum (Al)/TPA-ph-TPA and TPA-azo-TPA/ITO structures, Al top electrodes were thermally evaporated onto the film surface under 2 × 10^−6^ Torr through a shadow mask with a thickness of about 60 nm and an area of 0.20 mm^2^.

Fabrication of flexible memory devices: using 30 × 30 mm sized poly(ethylene terephthalate) (PET) as the flexible substrate, Al bottom electrodes were thermally evaporated onto the PET substrate under 2 × 10^−6^ Torr through a shadow mask with a thickness of about 100 nm and an area of 1 × 30 mm. Then, an 80 nm thickness organic layer was vacuum deposited onto the bottom Al electrodes as measured using the spectroscopic ellipsometer. The Al top electrodes were thermally evaporated onto the organic layer through a shadow mask with a thickness of about 100 nm and an area of 1 × 30 mm.

### Synthetic procedures

#### Synthesis of 1,2-bis(4-bromo-2,3,5,6-tetrafluorophenyl)diazene (1)

Compound 1 was prepared according to a previous method found in the literature.^24^ To a mixture of 4-bromo-2,3,5,6-tetrafluoroaniline (0.5 mmol) and NaI (1.0 mmol, 150.0 mg) in *t*-BuOH (3 mL), was added *tert*-butyl hypochlorite (1.0 mmol, 108.6 mg) under an N_2_ atmosphere at room temperature for 3 h. The mixture was stirred for the indicated time and quenched with aqueous sodium thiosulfate (1.0 M, 10 mL), and the solution was extracted with dichloromethane (DCM (CH_2_Cl_2_); 20 mL × 3). The combined organic extracts were dried over sodium sulfate and concentrated under vacuum to give the crude product. Purification using flash column chromatography on silica gel [eluent: CH_2_Cl_2_ in petroleum ether (PE)] gave the product. ^19^F-NMR [282 MHz, deuterated dimethylsulfoxide (DMSO-*d*_6_)] *d* (ppm): −131.23 (d, *J* = 16.0 Hz, 2F), −133.64 (d, *J* = 13.3 Hz, 2F), −147.70 (d, *J* = 16.0 Hz, 2F), −148.61 (d, *J* = 13.3 Hz, 2F); MS: calc'd for C_12_Br_2_F_8_N_2_ [M + H]^+^ 483.9370, found 483.8380. Anal. found for C_12_Br_2_F_8_N_2_: C, 29.98; Br, 32.84; N, 5.75.

#### Synthesis of 2,7-dibromo-1,3,4,6,8,9-hexafluorophenazine (2)

The synthesis method of 2 is similar to the method of synthesis for 1.

^19^F-NMR (282 MHz, DMSO-*d*_6_) *d* (ppm): −118.49 (d, *J* = 17.2 Hz, 2F), −125.15 (d, *J* = 18.3 Hz, 2F), −151.82–151.95 (m, 2F). MS: calc'd for C_12_Br_2_F_6_N_2_ [M + H]^+^ 443.8332, found 443.8040. Anal. found for C_12_Br_2_F_6_N_2_: C, 32.32; Br, 35.84; N, 6.27.

#### General procedure for the synthesis of TPA-azo-TPA and TPA-ph-TPA

A solution of 1 or 2 (0.409 g, 1 mmol), 4-(diphenylamino)phenylboronic acid (0.723 g, 2.5 mmol) and tetrakis(triphenylphosphine)palladium(0) (0.062 g, 0.05 mmol) dissolved in toluene (15 mL) was stirred in a two-necked flask under a N_2_ atmosphere for 30 min. To the reaction mixture, potassium carbonate (0.28 g, 2 mmol) in distilled water (15 mL) was added dropwise over a period of 20 min. The resulting solution was refluxed overnight at 80 °C. The reaction mixture was extracted with dichloromethane (DCM) and the organic layer was separated. The organic layer was evaporated with a rotary evaporator, and then the resulting powdery product was purified using column chromatography with DCM : PE (1 : 5) as the eluent and a crystalline solid was obtained.

#### Spectral data

##### 4′,4′′′-(Diazene-1,2-diyl)bis(2′,3′,5′,6′-tetrafluoro-*N*,*N*-diphenyl-[1,1′-biphenyl]-4-amine) (TPA-azo-TPA)

Yield 74%, ^1^H-NMR [400 MHz, deuterated chloroform (CDCl_3_)] *d* (ppm): 7.41–7.31 (m, 12H), 7.20–7.05 (m, 16H); ^13^C-NMR (100 MHz, CDCl_3_) *d* (ppm): 147.3, 146.9, 132.0, 131.0, 129.5, 129.4, 129.3, 125.5, 125.4, 124.9, 124.1, 123.4, 122.1, 121.3; ^19^F-NMR (376 MHz, CDCl_3_) *d* (ppm): 111.6, 127.1, 144.2, 149.6; TOF-MS: calc'd for C_48_H_28_F_8_N_4_ [M + H]^+^ 812.2186, found 812.2000; anal. found for C_48_H_28_F_8_N_4_: C, 70.65; H, 3.49; N, 6.92.

##### 4,4′-(perfluorophenazine-2,7-diyl)bis(*N*,*N*-diphenylaniline) (TPA-ph-TPA)

Yield: 82%, ^1^H-NMR (400 MHz, CDCl_3_) *d* (ppm): 7.70 (s, 2H), 7.54–7.52 (m, 4H), 7.34–7.33 (m, 6H), 7.24–7.18 (m, 12H), 7.15–7.13 (m, 4H); ^13^C-NMR (100 MHz, CDCl_3_) *d* (ppm): 146.4, 144.4, 131.0, 129.1, 128.9, 125.2, 123.7, 120.8, 104.5; ^19^F-NMR (376 MHz, CDCl_3_) *d* (ppm): 129.2, 130.9, 153.1; TOF-MS: calc'd for C_48_H_28_F_6_N_4_ [M + H]^+^ 774.2218, found 774.2210; anal. found for C_48_H_28_F_6_N_4_: C, 74.70; H, 3.66; N, 7.26.

## Results and discussion

### Thermal stability

**Fig. 1:**
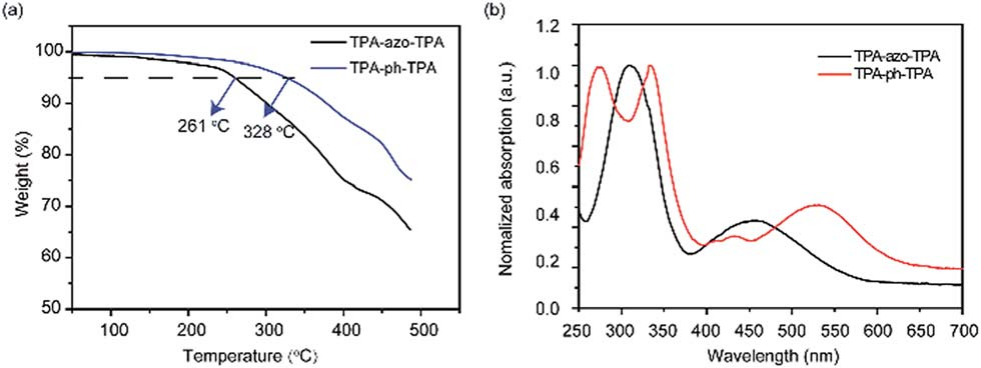
(a) TGA curves of TPA-azo-TPA, and TPA-ph-TPA at a heating rate of 10 °C min^−1^ under a N_2_ atmosphere; (b) UV-vis absorption spectrum of TPA-azo-TPA, and TPA-ph-TPA in DCM solution.

The molecules TPA-ph-TPA and TPA-azo-TPA can be obtained in moderate yields using a multistep Suzuki cross-coupling reaction. The thermal decomposition temperature of the TPA-azo-TPA is 261 °C, however, TPA-ph-TPA exhibits good thermal stability with a decomposition temperature above 300 °C (Fig. 1a). Compared with the currently reported azo-based small molecules,^25–27^ the stability of TPA-ph-TPA was good. This suggested that increasing the degree of conjugation of the molecular framework leads to the further improvement of the molecular heat resistance.

### Photophysical and electrochemical properties

The TPA-ph-TPA and TPA-azo-TPA have good solubility and so DCM was chosen as the solvent and a spin coating method was used to fabricate the film. Fig. 1b shows the optical absorption spectra of TPA-ph-TPA and TPA-azo-TPA nano-films on quartz substrates. The strong absorption bands at approximately 400–500 nm can be attributed to the n–π* transition (charge transfer) of the azobenzene.^28^ Compared with that of TPA-azo-TPA, the onset optical absorbance of TPA-ph-TPA exhibits a significant red-shift for 34 nm, which corresponds to the narrower energy band gap of the phenazine structure. The optical band gaps of the TPA-ph-TPA and TPA-azo-TPA molecules, estimated from the absorption edges of the films, were 2.03 eV and 2.15 eV, respectively. Therefore, increased conjugation with the phenothiazine structure gives rise to a ground state charge transfer complex in TPA-ph-TPA. This also suggests that the TPA-ph-TPA film forms an ordered stacking of the π-conjugation system, favouring an effective carrier migration.

**Fig. 2:**
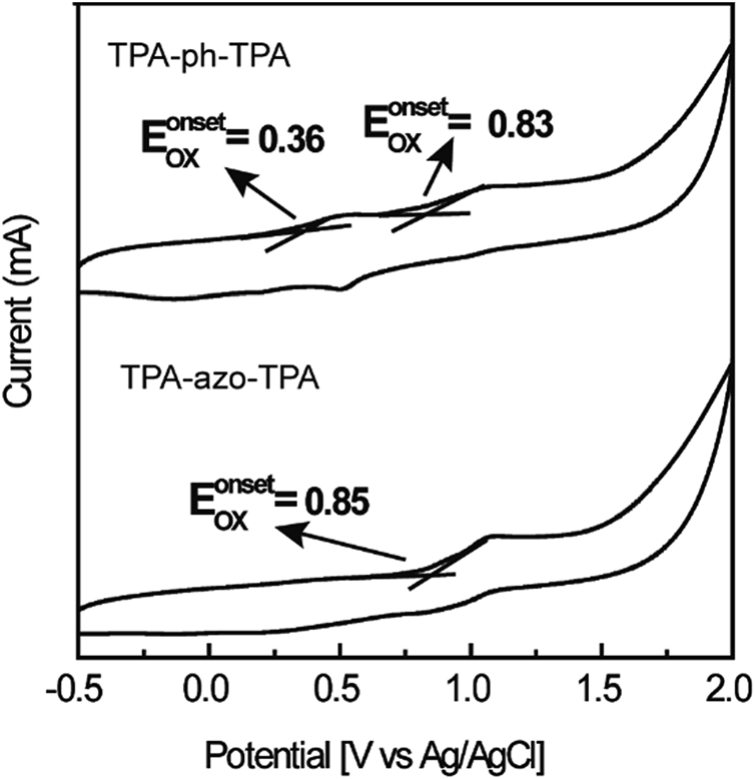
Cyclic voltammograms of TPA-azo-TPA and TPA-ph-TPA film were measured in 0.1 mmol L^−1^ TBAPF_6_/CH_3_CN solution with Ag/AgCl as reference electrode and platinum wire as the counter electrode. A scan rate of 100 mV s^−1^ was used.

Fig. 2 shows the cyclic voltammetry (CV) measurements of TPA-azo-TPA and TPA-ph-TPA on an ITO glass substrate in a 0.1 mol L^−1^ solution of tetrabutylammonium hexafluorophosphate (TBAPF_6_) in anhydrous acetonitrile (CH_3_CN) solution and measurements were taken with a scan rate of 100 mV s^−1^. The onset oxidation (*E*^onset^_ox_) of TPA-azo-TPA is approximately 0.85 V *versus* silver/silver chloride (Ag/AgCl). The onset oxidations (*E*^onset^_ox_) of TPA-ph-TPA were 0.35 V and 0.83 V *versus* Ag/AgCl, respectively. The oxidation potential onset of ferrocene *E*^onset^_Fe_ was 0.44 V *versus* Ag/AgCl in acetonitrile with bare ITO glass. The estimated highest occupied molecular orbital (HOMO) levels can be calculated from the *E*^onset^_ox_ using the following formula: HOMO = −[*E*^onset^_ox_ + 4.8 − *E*_Fc_] eV. The lowest unoccupied molecular orbital (LUMO) levels are not detectable using CV and so the values of the LUMO levels were estimated using the following formula: LUMO = [HOMO + *E*_g_] eV. The HOMO levels of TPA-azo-TPA and TPA-ph-TPA were −5.21 eV and −4.72 eV, respectively. The determined LUMO levels of TPA-azo-TPA and TPA-ph-TPA were −3.06 eV and −2.69 eV, respectively. TPA-ph-TPA has lower LUMO levels than TPA-azo-TPA, because of the charge transfer process, and this result was in agreement with the UV spectroscopic data.

### Morphology of the thin film

**Fig. 3:**
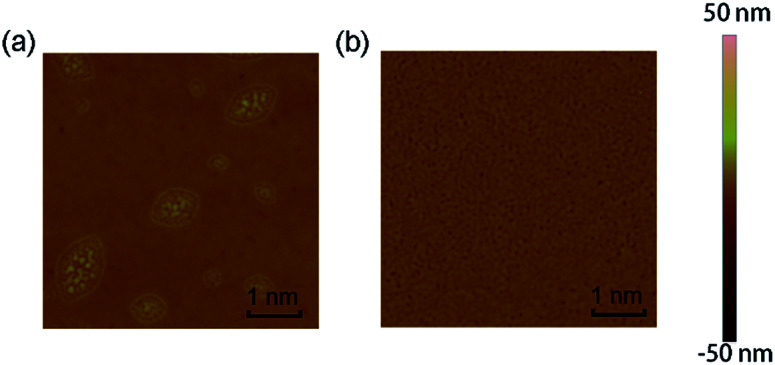
Tapping mode (5 μm × 5 μm) AFM phase of TPA-azo-TPA (a) and TPA-ph-TPA (b) film on ITO substrates.

To investigate the surface morphology and film microstructure, AFM measurements were made on the TPA-azo-TPA and TPA-ph-TPA film. As seen from the AFM height images (Fig. 3), both of the films showed a relatively smooth surface without obvious defects. The TPA-azo-TPA film clearly showed two-dimensional grains in its solid state at room temperature with uniform size and the surface root-mean-square (RMS) roughness was 1.8 nm. The TPA-ph-TPA film showed a denser needle morphology, and had a RMS roughness of 0.9 nm, which was smaller than that of TPA-azo-TPA. Therefore, the film quality was improved significantly.

### Current–voltage (*I*–*V*) characteristics of the memory devices

**Fig. 4:**
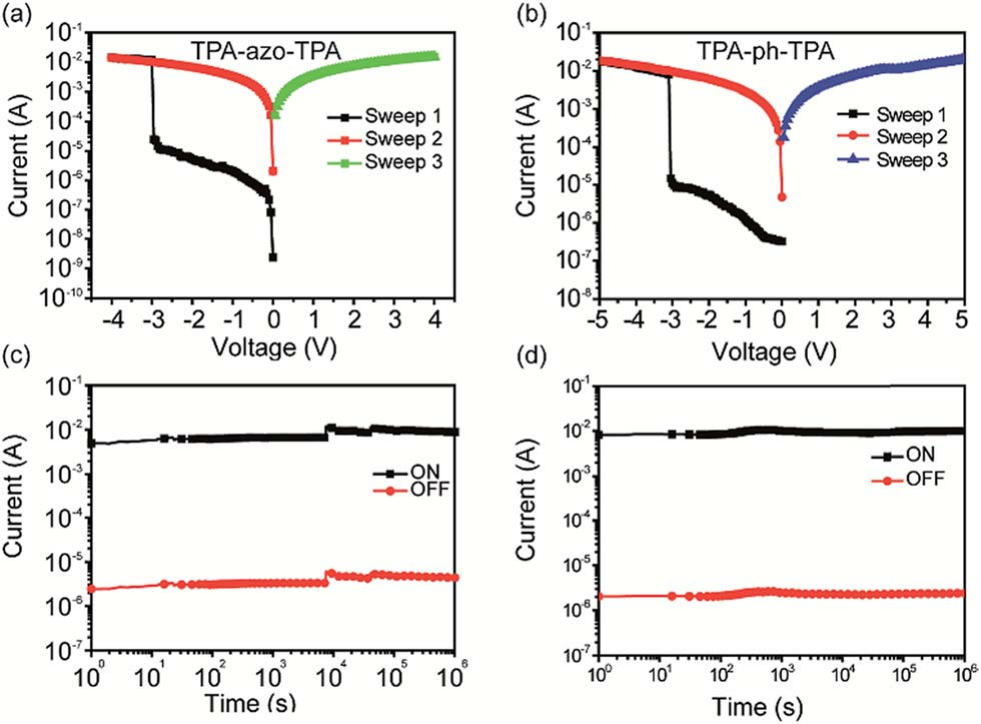
*I*–*V* characteristics of the small molecule-based memory devices: (a) Al/TPA-azo-TPA/ITO and (b) Al/TPA-ph-TPA/ITO; the effect of retention time of (c) the Al/TPA-azo-TPA/ITO and (d) the Al/TPA-ph-TPA/ITO memory devices under a constant stress of −1.0 V.

The memory effect of the TPA-azo-TPA devices was explored first, and the current–voltage (*I*–*V*) characteristics are shown in Fig. 4a. Under a negatively biased voltage sweep, the current in the fabricated Al/TPA-azo-TPA/ITO memory devices increased abruptly from 10^−5^ A to 10^−2^ A at around 3.0 V, indicating that the device has been switched from an OFF state to an ON state (sweep 1 of Fig. 4a). This transition can be defined as the “Write” process and an ON/OFF ratio of 10^3^ can be obtained. The device remained in the ON state, even after the power was turned off or during the subsequent voltage sweep (sweep 2). The ON state was maintained in the final sweep from 0 to −4 V (sweep 3). Therefore, the TPA-azo-TPA-based device exhibited a typical nonvolatile write once read many (WORM) memory behavior. Compared with the Al/TPA-azo-TPA/ITO devices, the Al/TPA-ph-TPA/ITO devices exhibited much smoother *I*–*V* characteristics during the switching process. As shown in Fig. 4b, the Al/TPA-ph-TPA/ITO memory cell was in the OFF state. When a negatively biased potential sweep from 0 to −4 V was applied (sweep 1 in Fig. 4b), the current increased consecutively from 10^−6^ A to 10^−2^ A, indicating that the device had been set to the ON state by the negative forward sweep. The device remained in the ON state during the subsequent voltage sweep (sweep 2). In the following positive sweep from 0 to 4 V (sweep 3), the device remained in the ON state. The value of the ON/OFF ratio for TPA-ph-TPA-based devices was 10^4^. The TPA-azo-TPA-based device also showed a typical nonvolatile WORM memory performance. Both of these two devices have potential applications for non-volatile data storage. Compared with the Al/TPA-azo-TPA/ITO device, the TPA-ph-TPA-based device has a higher ON/OFF ratio, which is helpful for avoiding false programming and error readout problems.

In order to exclude the possibility of the influence of metal filaments in the memory behaviours, sandwich structured memory devices: Al/LiF/small molecules/ITO were fabricated using the same conditions. The LiF layer (5 nm) acted as a buffer layer to prevent the Al nanoparticles penetrating into the active films. The *I*–*V* results indicated the WORM memory behaviours of TPA-azo-TPA and TPA-ph-TPA based memory devices, as shown in Fig. S1 and S2 (ESI†), which demonstrated that the WORM memory behaviours were not dependent on the metal filament.

The retention times of the ON state and the OFF state of the Al/TPA-azo-TPA/ITO and Al/TPA-ph-TPA/ITO device is shown in Fig. 4c and d. Under a constant stress of −1 V, TPA-ph-TPA-based devices were observed to have more stability and no significant degradation in current for any of the states at least 10^6^ s during the readout test. The long-term stability of two molecular-based devices under air and humidity conditions was measured, and the results of this are shown in Fig. S3 and S4 (ESI†). The test results show that the TPA-ph-TPA-based devices have better long-term stability than the TPA-ph-TPA-based devices. This may be because the phenazine group is more stable than the azobenzene group in air and humidity conditions.

Because of the rigid planarity of the glass substrates and the brittleness of the ITO electrodes, which limits its application on flexible devices. Flexible substrates such as PET were used to replace the ITO glasses and to fabricate flexible memory device.^29–31^ The specific fabricated method is described in the Experimental section. The flexible memory device can be bent under different conditions and the degree of bending was defined by the distance (*D*) between the ends of the arc. The top and bottom Al electrodes of this flexible device were aligned perpendicularly to each other, and the voltage bias was applied to the memory devices at the end of the top Al electrode (cathode).

**Fig. 5:**
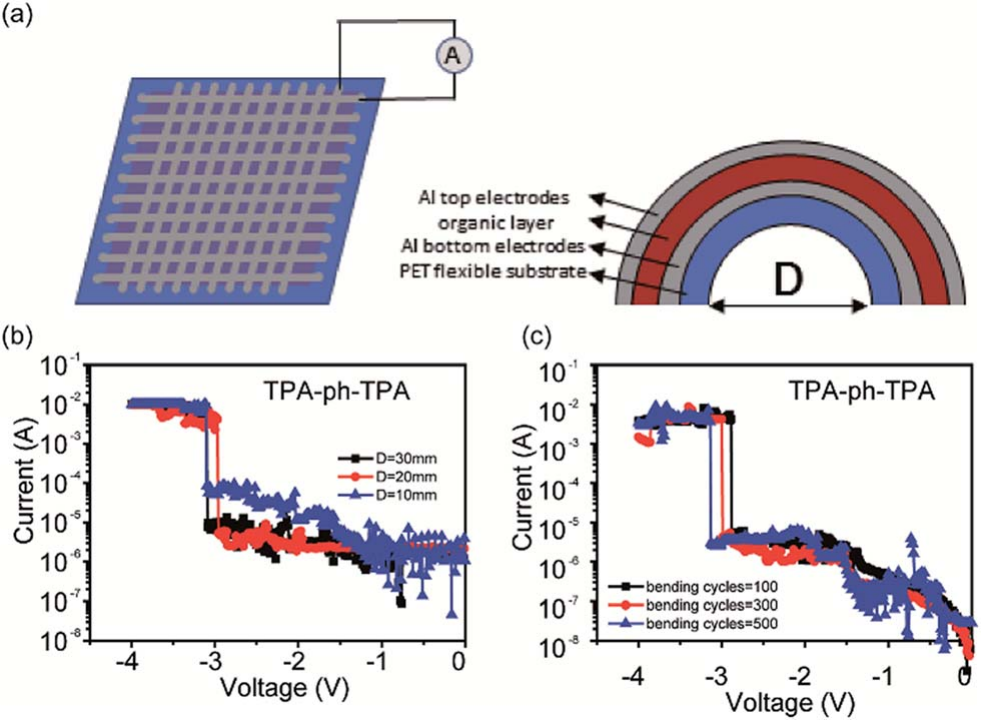
(a) The model of the sandwich structure flexible memory device consisting of an active layer between the top and bottom Al electrodes (left), the calculation of the bending degree of the flexible memory device (right); (b) the memory effects of the TPA-ph-TPA-based flexible memory devices under different degrees of bending and the memory device can maintain the memory effect when the distance between the ends of the arc reaches 10 mm. (c) The TPA-ph-TPA-based device can maintain memory performance within 500 bending cycles in the maximum bending condition.

To confirm the feasibility of flexible memory devices, the sandwich structured flexible memory device with a 10 (word line) × 10 (bit line) crossbar array is shown in Fig. 5a. The electric properties were measured under different bending conditions. For the TPA-azo-TPA-based flexible memory device (Fig. S5; ESI†), the storage units of different regions could show stable binary memory behavior even when the distance between the ends of the arc bending was 20 mm. When *D* reached 10 mm, the storage units of the TPA-azo-TPA-based flexible memory device became unstable. Additionally, the *I*–*V* curves of the Al/TPA-ph-TPA/Al/PET structured memory device were also measured to test its flexibility. As shown in Fig. 5b, the device also maintained stable binary memory characteristics when *D* was 10 mm. The electrical characteristics of each curve are summarized from results obtained from 20 different cells under each bending condition.

The *I*–*V* characteristics of flexible memory device over bending cycles of 500 times were also measured. The degree of bending was from the flat state to the maximum degree of bending (*D* = 10 mm) and as the number of bending cycles increased, the TPA-azo-TPA-based flexible memory device cannot maintain stable binary memory behaviour, as shown in Fig. S6 (ESI†). However, the TPA-ph-TPA-based flexible memory device showed stable binary memory behaviour up to 500 bending cycles of, as shown in Fig. 5c. In addition, the threshold voltage is a little larger than the previous bending and therefore shows a little shifting after several cycles of bending. This is probably because of an increase in the number of bending times, and the film cannot withstand the mechanical stretch and the ordered molecular stacking might be somehow damaged. Thus, the charges may have difficulty in moving through the films and much energy is needed to overcome the barrier. For bending cycles above 500, the TPA-ph-TPA-based memory device showed no visible binary memory behaviour.

### Memory mechanism

**Fig. 6:**
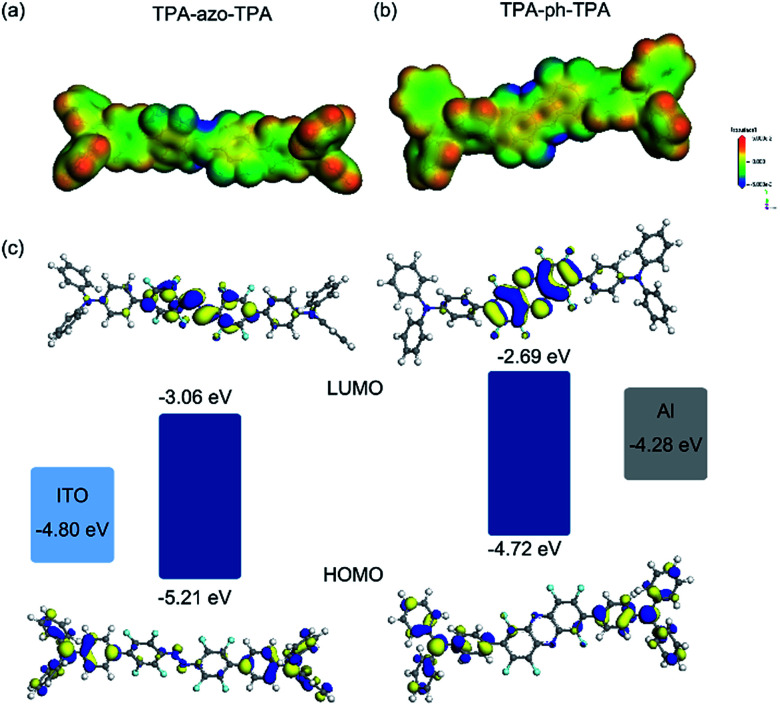
Simulated electrostatic potential (ESP) plots of TPA-azo-TPA (a) and TPA-ph-TPA (b); HOMO, LUMO and energy level diagrams for the ITO/TPA-azo-TPA and TPA-ph-TPA/Al devices (c).

To better understand the electronic process occurring inside the thin film, theoretical calculations were performed using density-functional program DMol_3_.^32,33^ The hybrid functional B3LYP,^34,35^ together with a double numerical plus polarization (DNP) basis set, was used. Comparing the *E*_gap_ of TPA-azo-TPA and TPA-ph-TPA, the TPA-ph-TPA has a lower *E*_gap_, which makes charge carrier migration easier. The molecular orbitals (HOMO, LUMO) and the electrostatic surface potential (ESP) of TPA-azo-TPA and TPA-ph-TPA molecules were plotted, and are shown in Fig. 6.

To better understand the charge carrier behavior of TPA-azo-TPA and TPA-ph-TPA based devices, the work functions (F) of Al top and ITO bottom electrodes were compared with molecular HOMO and LUMO orbital. The energy barrier between *F* of the ITO (−4.8 eV) and the HOMO energy level was 0.35–0.41 eV, which is much lower than the energy barrier of 1.21–1.39 eV between the *F* of the Al (−4.3 eV) and the LUMO energy level. This suggests that hole injection from ITO into the HOMO of TPA-azo-TPA or TPA-ph-TPA (corresponding to ITO as the anode) is easier than electron injection from Al into the LUMO of TPA-azo-TPA or TPA-ph-TPA. Thus, TPA-azo-TPA and TPA-ph-TPA are p-type materials and holes predominate the conduction process (as shown in the inset of Fig. 6).

The ESP plots of TPA-azo-TPA and TPA-ph-TPA molecules both show an open channel along the molecular backbone with a continuous positive electrostatic potential, providing a path to allow charge carrier migration. For the TPA-azo-TPA molecule, electrons transit readily from the HOMO orbital to the LUMO orbital under an external electric field, forming the locally excited state. As a result, a charge-transfer interaction can occur in the TPA-azo-TPA molecule between the electron donor moieties and the electron acceptor moieties. For the TPA-ph-TPA molecule, a similar charge transfer process is also formed. However, the phenazine conjugation structure enhances the electron-withdrawing ability of the acceptor moiety, so the HOMO and LUMO orbitals of TPA-ph-TPA molecules show more obvious separation in the ground state, with HOMO mostly localizing on the donor areas and LUMO mostly localizing on the acceptor areas. It was speculated that the mechanism of the memory performance of the TPA-azo-TPA and TPA-ph-TPA devices could be the electric field induced charge transfer effect. Without an external electric field, the electrons in the TPA-azo-TPA and TPA-ph-TPA molecules were stable, and the as-fabricated device was in the OFF state. When a negative bias is applied, the charge transport pathways will form, and will switch the Al/TPA-azo-TPA or TPA-ph-TPA/ITO device from the OFF state to the ON state. However, the phenazine conjugation structure can significantly enhance the electron-withdrawing ability of the molecule, resulting in a stronger intramolecular charge transport in TPA-ph-TPA. It has been reported that changing the charge transfer ability of the D–A molecules can tune memory effects. So the better performance (larger ON/OFF ratio and more stability with each electric conductive state) of the TPA-ph-TPA-based device could be because of the stronger intramolecular charge transfer effect. The nonvolatile nature of the ON state is because of the intensive electron delocalization in the acceptor moieties which stabilized the conductive charge transfer state.

## Conclusions

In conclusion, new materials based on fluorine substituted azobenzene-π-triphenylamine derivatives (TPA-azo-TPA) and fluorine substituted phenazine-π-triphenylamine derivatives (TPA-ph-TPA) were synthesized. The photophysical, electrochemical properties and memory behaviors of these donor–π–acceptor molecules were investigated and compared. This comparative study of tuning the properties of the conjugated D–A–D molecules using an aromatic acceptor may be an alternative approach for the design and study of future high-performance memory devices based on new materials.

## Conflicts of interest

There are no conflicts to declare.

## Supplementary Material

RA-008-C7RA11932A-s001
