# Supplementary material for: Changing molecular conjugation with a phenazine acceptor for improvement of small molecule-based organic electronic memory performance
Source: RSC Adv. 2018 Jan 3;8(2):805–11. doi: 10.1039/c7ra11932a (PMC9076932; doi:10.1039/c7ra11932a)
Supplement: RA-008-C7RA11932A-s001 [file RA-008-C7RA11932A-s001.pdf]

Electronic Supplementary Material (ESI) for RSC Advances.
This journal is © The Royal Society of Chemistry 2017

Supporting Information

Changing molecular conjugation with phenazine acceptor for small molecule-based organic electronic memory performance

*Quan Liu, *[a] Caibin Zhao,[a] Guanghui Tian[a] and Hongguang Ge[a]*

a. Shaanxi Province Key Laboratory of Catalytic Foundations and Applications, School of Chemical and Environmental Science, Shaanxi University of Technology, Hanzhong, 723001, China

Tel: +86 916 2641660; E-mail: liuq@snut.edu.cn

Contents

MS and NMR spectra of compounds……………………………………………………S2-S6

The I–V characteristics of the memory devices use LiF as block layer………….S7

The long-term stability of the memory devices under ambient condition………..S8

The memory behavior of the flexible memory devices under different bending conditions……………………………………………………………………………..S9

^{19}F NMR of compound 1

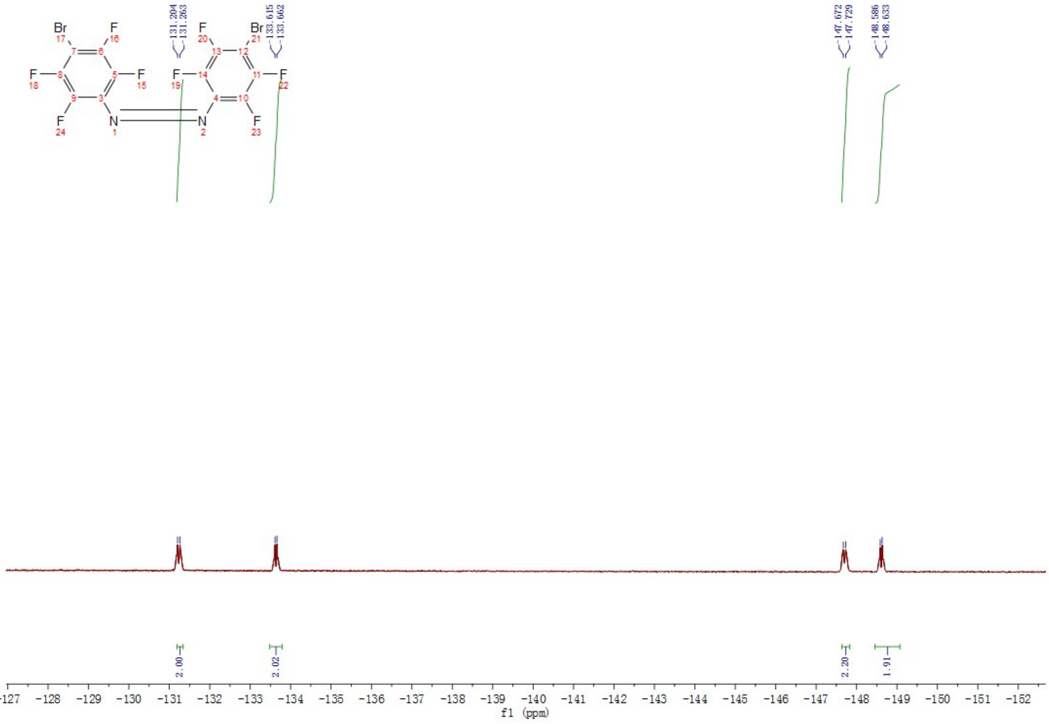

^{19}F NMR of compound 2

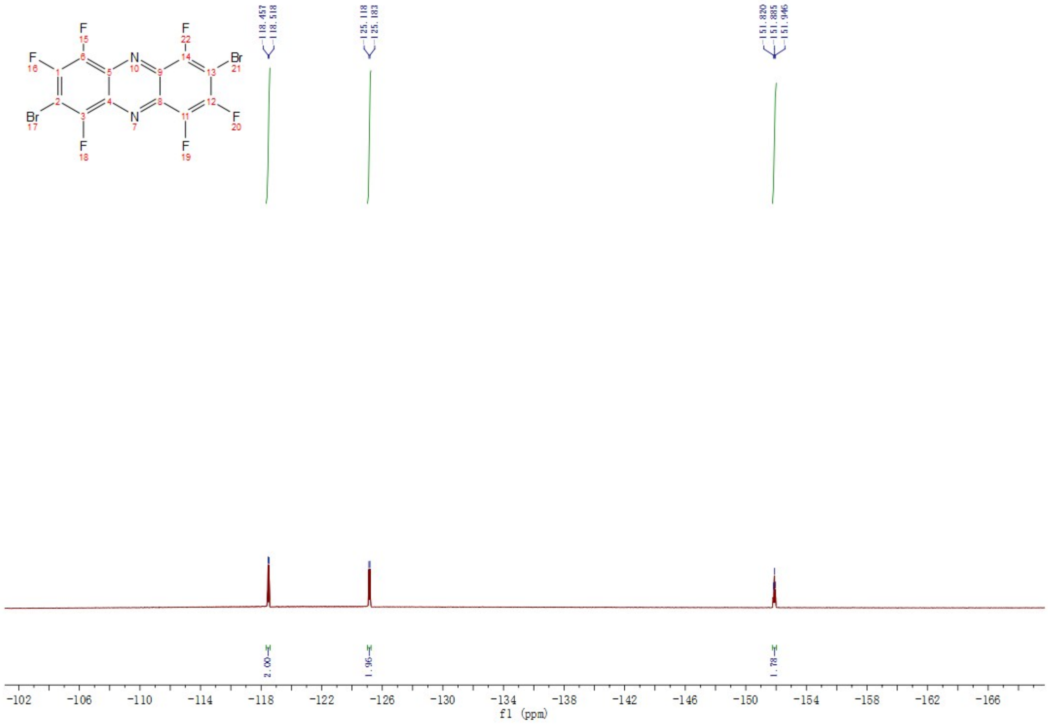

^1H NMR of compound ***TPA-azo-TPA***

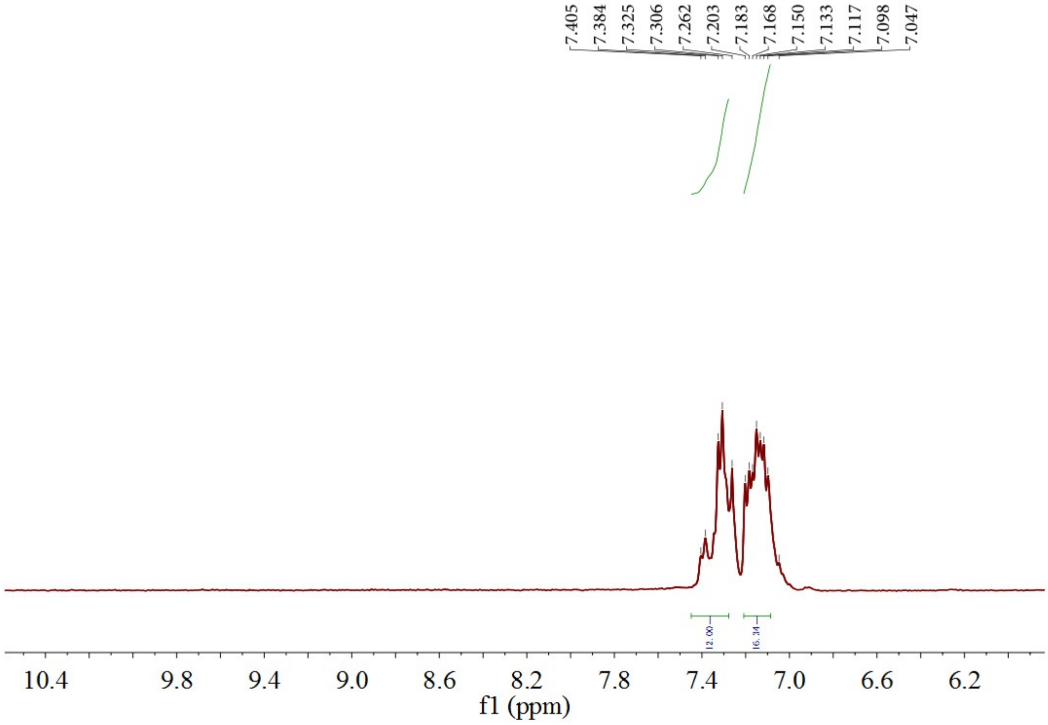

^{13}C NMR of compound ***TPA-azo-TPA***

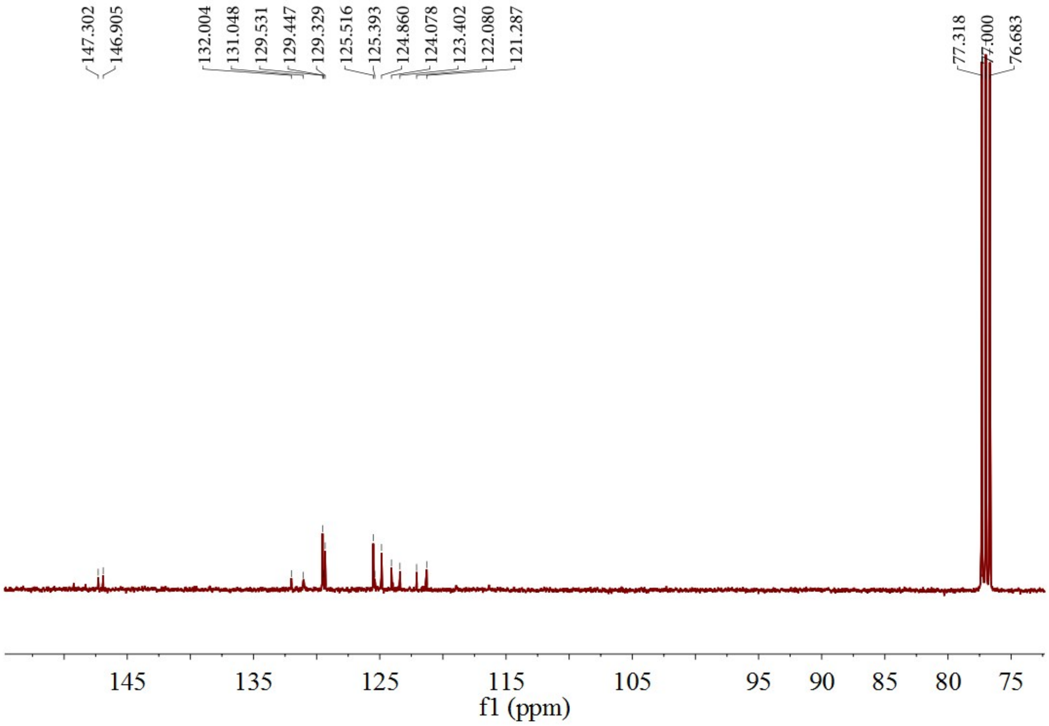

^{19}F NMR of compound ***TPA-azo-TPA***

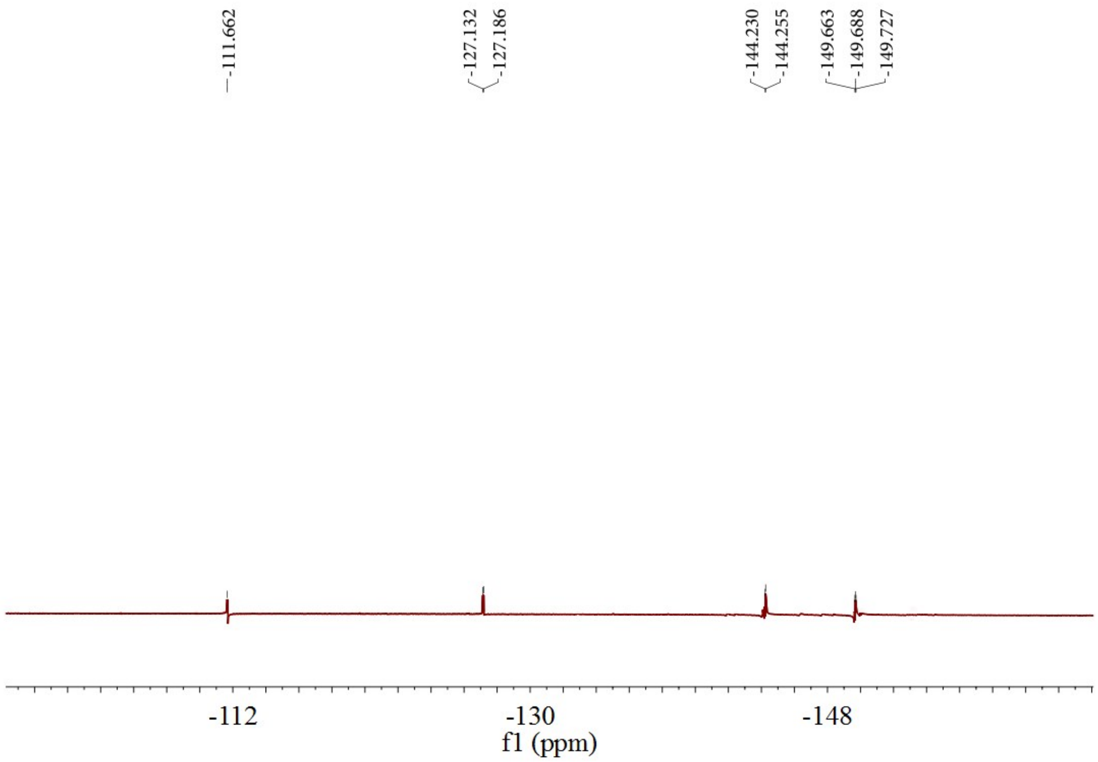

MS of compound ***TPA-azo-TPA***

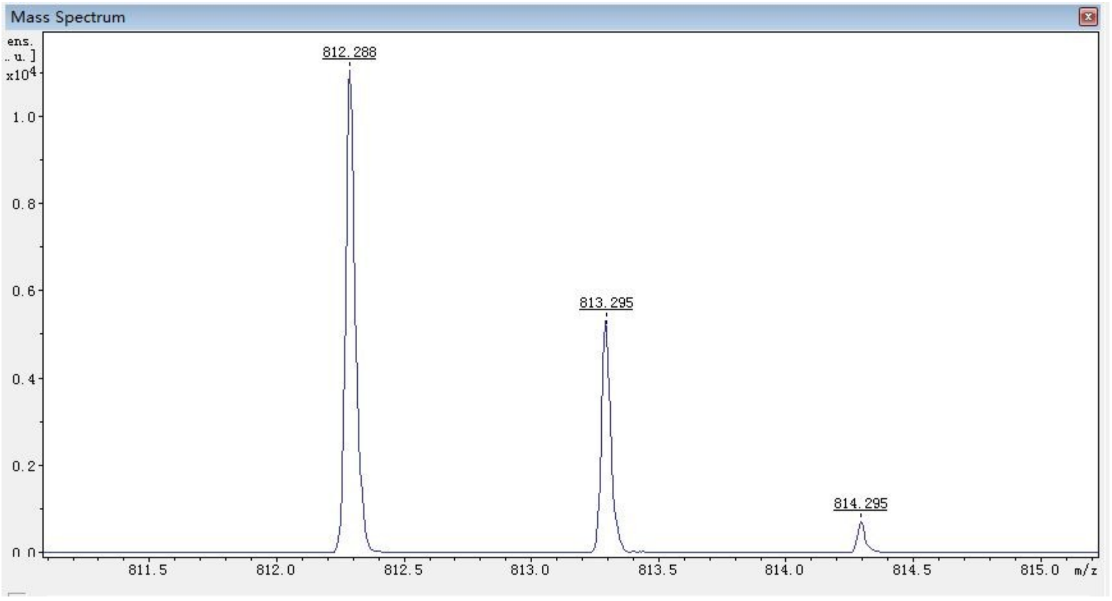

^{1}H NMR of compound ***TPA-ph-TPA***

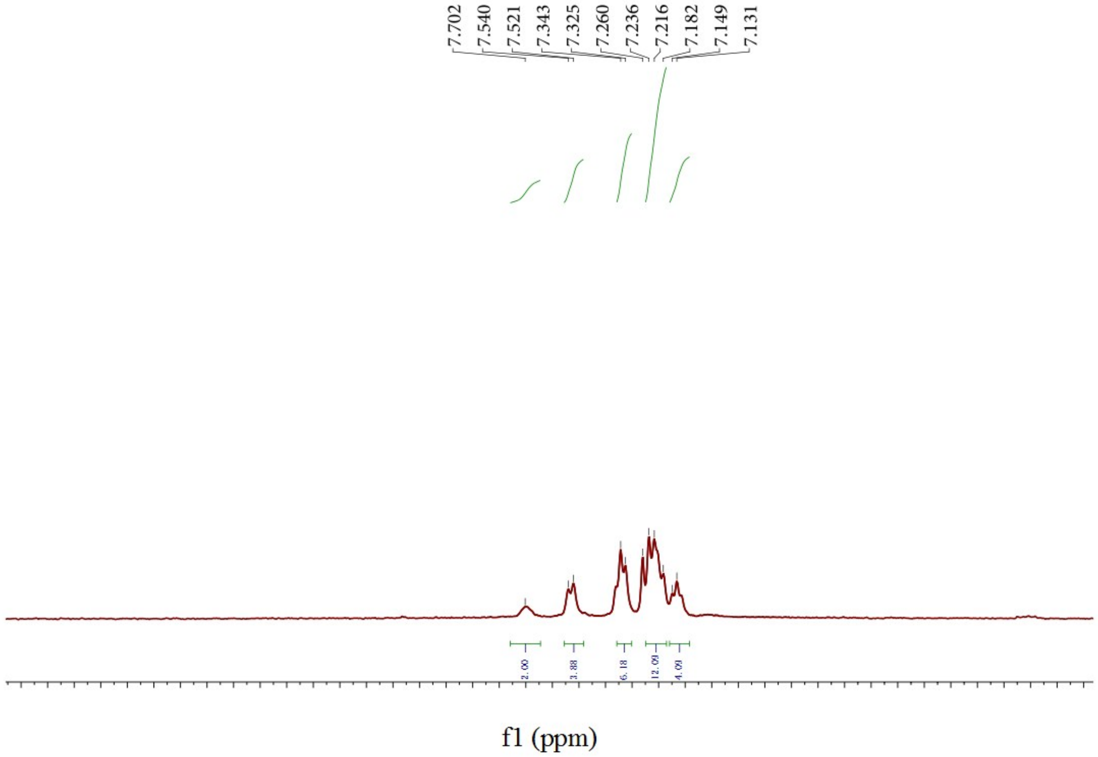

^{13}C NMR of compound ***TPA-ph-TPA***

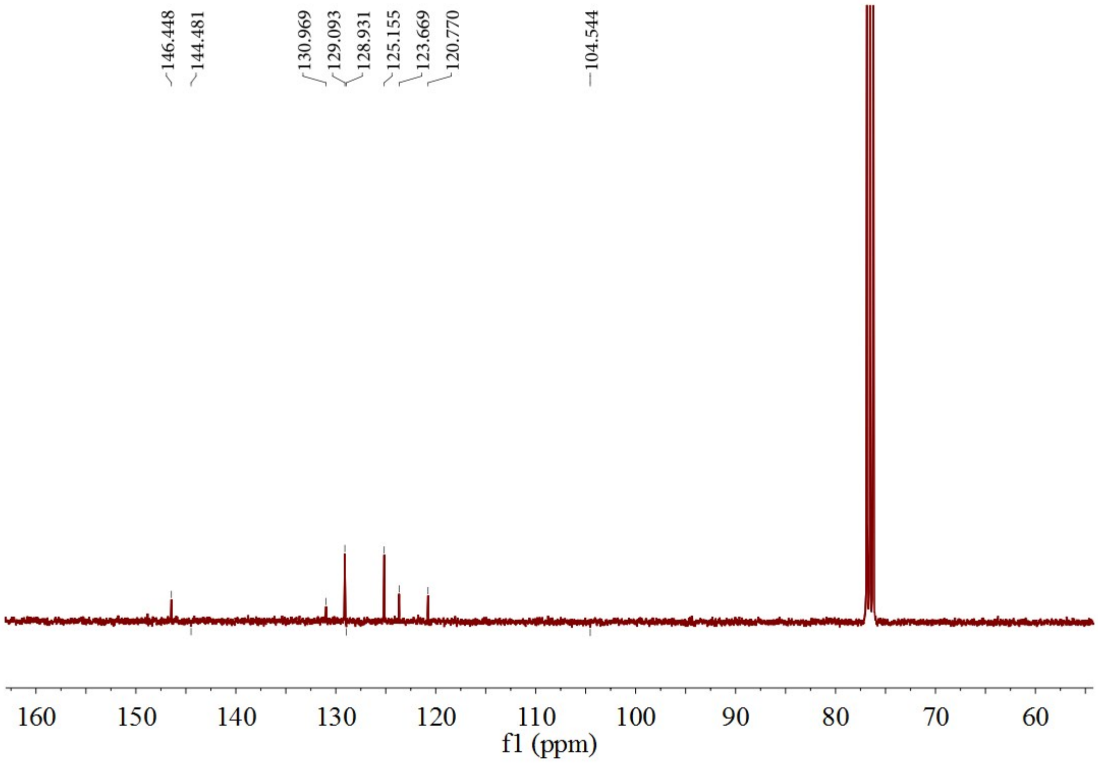

^{19}F NMR of compound ***TPA-ph-TPA***

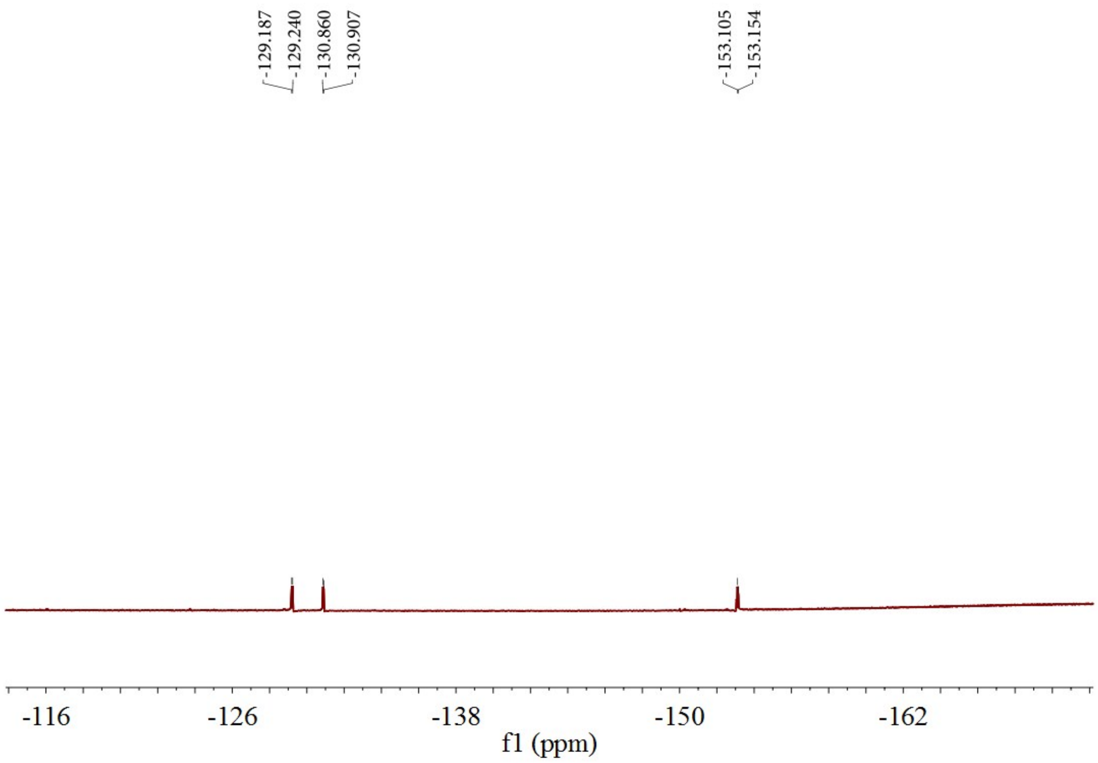

MS of compound ***TPA-ph-TPA***

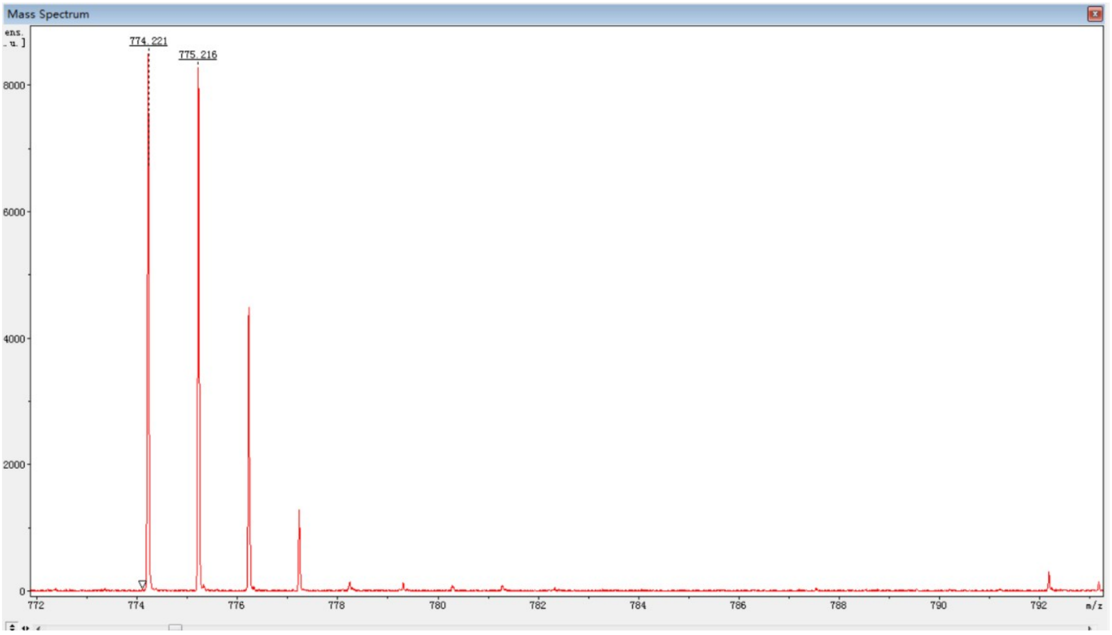

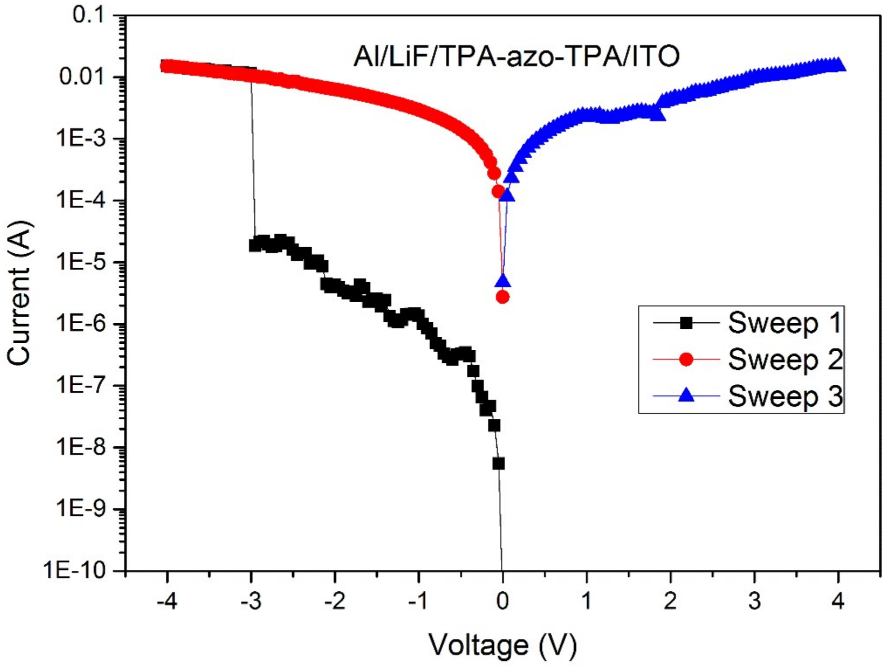

Figure S1 I-V characteristics of Al /LiF (5 nm)/ **TPA-azo-TPA** / ITO memory devices.

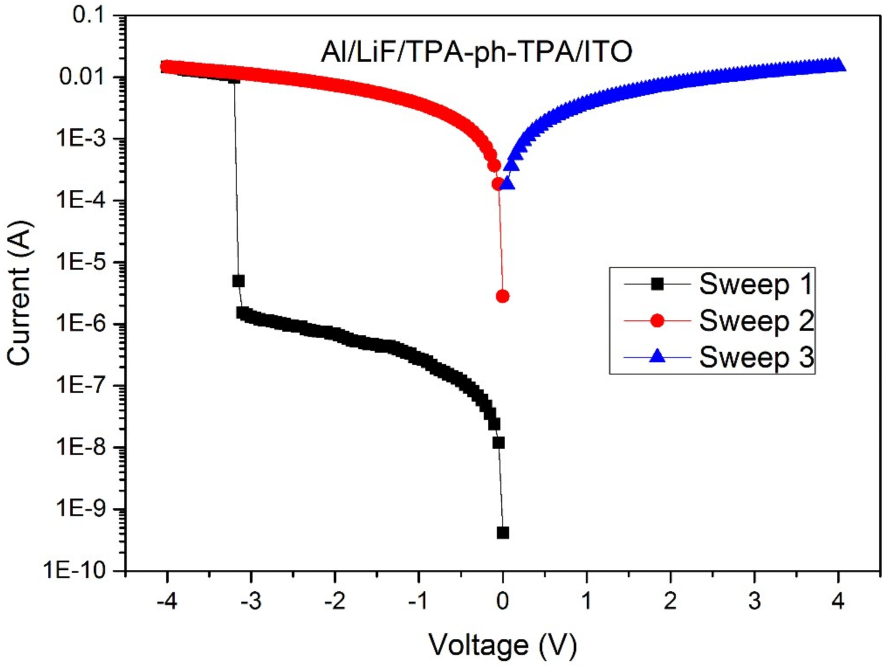

Figure S2 I-V characteristics of Al /LiF (5 nm)/ **TPA-ph-TPA** / ITO memory devices.

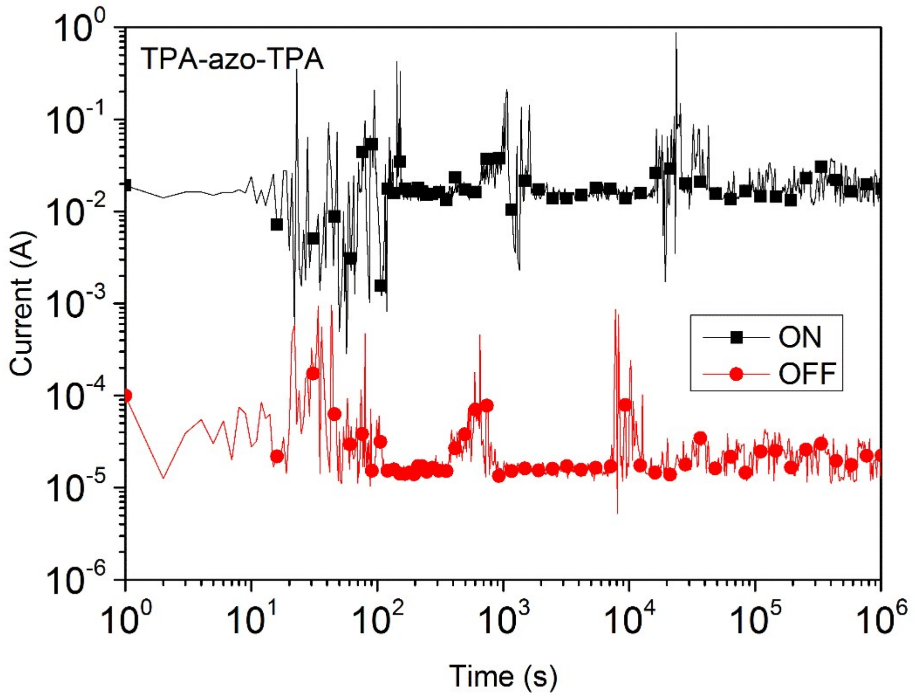

Figure S3 The effect of retention time of Al/ **TPA-azo-TPA** /ITO-based memory device under a constant stress of -1.0 V in air and humidity condition.

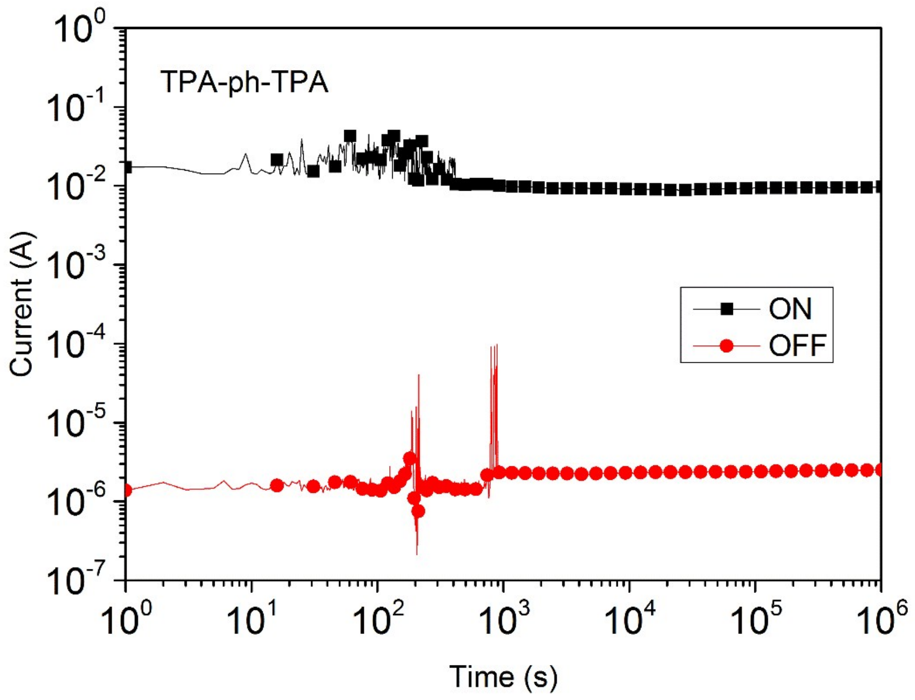

Figure S4 The effect of retention time of Al/ **TPA-ph-TPA** /ITO-based memory device under a constant stress of -1.0 V in air and humidity condition.

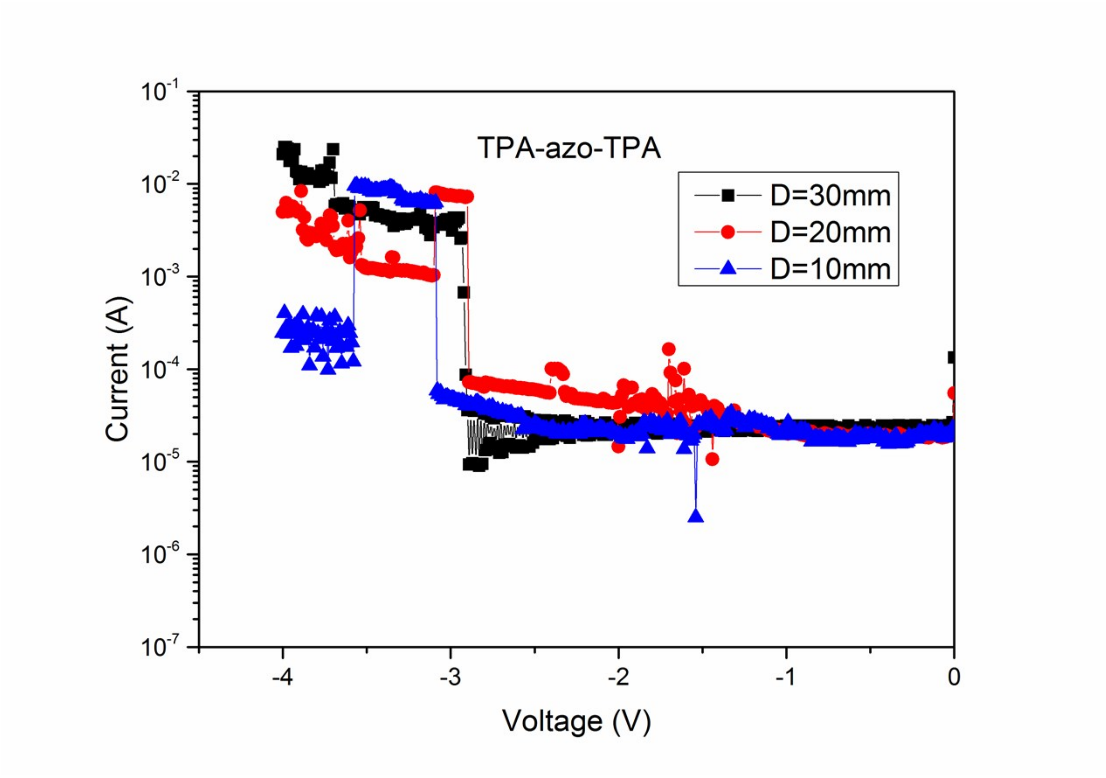

Figure S5 The memory effects of the Al/ **TPA-azo-TPA** /Al-based flexible memory devices in different bending degrees. the distance between the end of the arc define as (D).

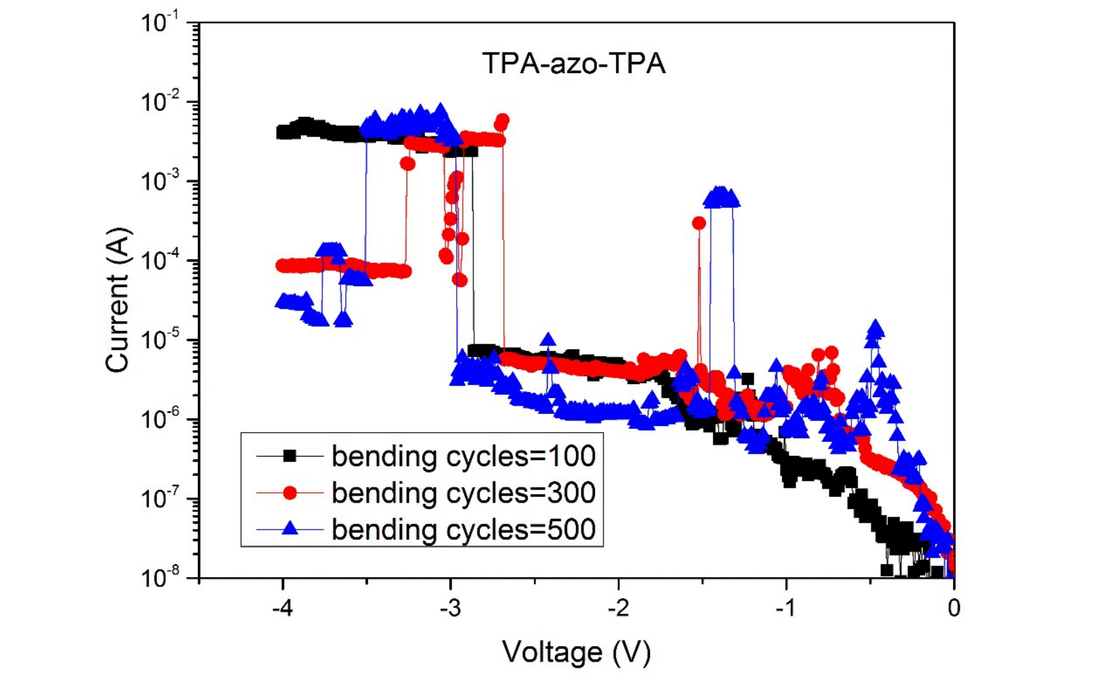

Figure S6 The memory effects of the Al/ **TPA-azo-TPA** /Al-based flexible memory devices under different bending cycles in the maximum bending condition.
